# Prognostic factors in patients progressing after cisplatin-based chemotherapy for malignant non-seminomatous germ cell tumours

**DOI:** 10.1038/sj.bjc.6690534

**Published:** 1999-07

**Authors:** S D Fosså, S P Stenning, A Gerl, A Horwich, P I Clark, P M Wilkinson, W G Jones, M V Williams, R T Oliver, E S Newlands, G M Mead, M H Cullen, S B Kaye, G J S Rustin, P A Cook

**Affiliations:** Department of Medical Oncology and Radiotherapy, The Norwegian Radium Hospital, Montebello, Oslo, 0310, Norway; MRC Cancer Trials Office, Cambridge, UK; Department of Medicine III, Klinikum Großhadern, University of Munich, Germany; Royal Marsden Hospital, Sutton, UK; Clatterbridge Hospital, Wirral, UK; Christie Hospital, Manchester, UK; Cookridge Hospital, Leeds, UK; Addenbrookes Hospital, Cambridge, UK; Royal London and St. Bartholomew's Hospital, London, UK; Charing Cross Hospital, London, UK; Royal South Hants Hospital, Southampton, UK; Queen Elizabeth Hospital, Birmingham, UK; Western Infirmary, Glasgow, UK; Mount Vernon Hospital, Northwood, UK

**Keywords:** germ cell malignancy, relapse, cisplatin-based chemotherapy, survival

## Abstract

The aim of this study was to define prognostic parameters for survival in patients with malignant germ cell tumours progressing after platinum-based induction chemotherapy with or without surgery. A total of 164 progressing patients (testicular: 83%, extragonadal: 17%) were identified out of 795 patients treated with platinum-based induction chemotherapy for metastatic germ cell malignancy with or without surgery. ‘Progressive disease’ included patients who had progressed after a previous partial or complete remission as well as patients who failed primary therapy. Salvage chemotherapy consisted of ‘conventional’ platinum-based chemotherapy. Prognostic factors for survival were assessed by uni- and multivariate analyses. The resulting prognostic model was validated in an independent data set of 66 similar patients. For all 164 patients the median time from start of induction chemotherapy to progression was 10 months (range: 0–99). Thirty-eight (23%) patients relapsed after 2 years. The 5-year survival rate for all progressing patients was 30% (95% confidence interval 23–38%). In the univariate analysis the following factors most importantly predicted a poor prognosis: progression-free interval < 2 years: initial poor prognosis category (MRC criteria), < CR to induction chemotherapy, initial treatment early in the 1980s and treatment given at a ‘small’ centre. Three prognostic factors remained in the multivariate analysis: progression-free interval, response to induction treatment and the level of serum human chronic gonadotrophin (hCG) and alpha fetoprotein (AFP) at relapse. One hundred and twenty-four patients could be classified on the basis of these characteristics, Those patients with progression-free interval < 2 years, < CR to induction chemotherapy and high markers at relapse (AFP >100 kU l^−1^ or hCG >100 IU l^−1^) formed a poor prognosis group of 30 patients, none of whom survived after 3 years. Patients with at most two of these three risk factors formed a good prognosis group of 94 patients (76%) with a 47% (37–56%) 5-year survival. Thirty-eight patients from the good prognosis group with a progression-free interval of >2 years had a 2-year survival of 74% (60–88%) and 5-year survival of 61%. These prognostic groups were validated in the independent data set, in which 5-year survival rates in the good and poor risk groups were 51% and 0% respectively. One-third of patients progressing during or after platinum-based induction chemotherapy for metastatic germ cell malignancy may be cured by repeated ‘conventional’ platinum-based chemotherapy. Good prognosis parameters are: progression-free interval of > 2 years, CR to induction treatment and normal or low serum markers at relapse (hCG < 100 IU l^−1^ and AFP < 100 kU l^−1^). The results of high-dose salvage chemotherapy should be interpreted on the background of these prognostic factors. © 1999 Cancer Research Campaign


*British Journal of Cancer* (1999) **80**(9), 1392–1399
© 1999 Cancer Research Campaign
Article no. bjoc.1999.0534

### Prognostic factors in patients progressing after cisplatin-based chemotherapy for malignant non-seminomatous germ cell tumours

**SD Fosså[1], SP Stenning[2], A Gerl[3], A Horwich[4], PI Clark[5], PM Wilkinson[6], WG Jones[7], MV Williams[8], RT Oliver[9], ES Newlands[10], GM Mead[11], MH Cullen[12], SB Kaye[13], GJS Rustin[14] and PA Cook[2]**

[1]Department of Medical Oncology and Radiotherapy, The Norwegian Radium Hospital, Montebello, 0310 Oslo, Norway; [2]MRC Cancer Trials Office, Cambridge, UK; [3]Department of Medicine III, Klinikum Großhadern, University of Munich, Germany; [4]Royal Marsden Hospital, Sutton, UK; [5]Clatterbridge Hospital, Wirral, UK; [6]Christie Hospital, Manchester, UK; [7]Cookridge Hospital, Leeds, UK; [8]Addenbrookes Hospital, Cambridge, UK; [9]Royal London and St. Bartholomew's Hospital, London, UK; [10]Charing Cross Hospital, London, UK; [11]Royal South Hants Hospital, Southampton, UK; [12]Queen Elizabeth Hospital, Birmingham, UK; [13]Western Infirmary, Glasgow, UK and [14]Mount Vernon Hospital, Northwood, UK

**Summary** The aim of this study was to define prognostic parameters for survival in patients with malignant germ cell tumours progressing after platinum-based induction chemotherapy with or without surgery. A total of 164 progressing patients (testicular: 83%, extragonadal: 17%) were identified out of 795 patients treated with platinum-based induction chemotherapy for metastatic germ cell malignancy with or without surgery. 'Progressive disease' included patients who had progressed after a previous partial or complete remission as well as patients who failed primary therapy. Salvage chemotherapy consisted of 'conventional' platinum-based chemotherapy. Prognostic factors for survival were assessed by uni- and multivariate analyses. The resulting prognostic model was validated in an independent data set of 66 similar patients. For all 164 patients the median time from start of induction chemotherapy to progression was 10 months (range: 0–99). Thirty-eight (23%) patients relapsed after 2 years. The 5-year survival rate for all progressing patients was 30% (95% confidence interval 23–38%). In the univariate analysis the following factors most importantly predicted a poor prognosis: progression-free interval < 2 years: initial poor prognosis category (MRC criteria), < CR to induction chemotherapy, initial treatment early in the 1980s and treatment given at a 'small' centre. Three prognostic factors remained in the multivariate analysis: progression-free interval, response to induction treatment and the level of serum human chronic gonadotrophin (hCG) and alpha fetoprotein (AFP) at relapse. One hundred and twenty-four patients could be classified on the basis of these characteristics, Those patients with progression-free interval < 2 years, < CR to induction chemotherapy and high markers at relapse (AFP >100 kU $l^{-1}$ or hCG >100 IU $l^{-1}$) formed a poor prognosis group of 30 patients, none of whom survived after 3 years. Patients with at most two of these three risk factors formed a good prognosis group of 94 patients (76%) with a 47% (37–56%) 5-year survival. Thirty-eight patients from the good prognosis group with a progression-free interval of >2 years had a 2-year survival of 74% (60–88%) and 5-year survival of 61%. These prognostic groups were validated in the independent data set, in which 5-year survival rates in the good and poor risk groups were 51% and 0% respectively. One-third of patients progressing during or after platinum-based induction chemotherapy for metastatic germ cell malignancy may be cured by repeated 'conventional' platinum-based chemotherapy. Good prognosis parameters are: progression-free interval of > 2 years, CR to induction treatment and normal or low serum markers at relapse (hCG < 100 IU $l^{-1}$ and AFP < 100 kU $l^{-1}$). The results of high-dose salvage chemotherapy should be interpreted on the background of these prognostic factors.

**Keywords:** germ cell malignancy; relapse; cisplatin-based chemotherapy; survival

Cisplatin-based chemotherapy represents an effective treatment for the majority of patients with advanced malignant germ cell tumour. However, about 20% of the patients progress during or after such chemotherapy and require salvage treatment (Mead et al, 1992). A variety of salvage treatments have been established which, in addition to platinum, usually include cytotoxic agents to which the patient has not been exposed previously, such as ifosfamide (Loehrer, et al, 1986), vinblastine (Loehrer et al, 1998, doxorubicin (Lederman and Garnick, 1986), methotrexate (Levi et al, 1990), actinomycin D (Levi et al, 1990) and paclitaxel (Motzer et al, 1994).

*Received 24 September 1998*
*Revised 4 January 1999*
*Accepted 21 January 1999*

*Correspondence to:* SD Fossa

High-dose chemotherapy (Broun et al, 1992; Siegert et al, 1994; Beyer et al, 1996; Margolin et al, 1996) with stem cell support is increasingly accepted as the treatment of choice for patients progressing during or after primary chemotherapy for germ cell malignancy. Disease-free survival rates of 40–50% have been reported after such treatment which seems superior to the

20% disease-free survival rates obtained with 'conventional' chemotherapy schedules. Although the toxicity of high-dose chemotherapy has decreased with increasing experience there remains a considerable risk of severe and even lethal toxicity associated with the treatment approach in these patients. Therefore, until the superiority of a high-dose approach as salvage treatment has been demonstrated in randomized trials, patients should not be subjected to such treatment unnecessarily. It is thus important to identify prognostic factors for salvage treatment for patients with disease activity of their germ cell malignancy during or after induction chemotherapy.

**Table 1** Pretreatment characteristics and induction treatment

| Characteristic | Group | Number of patients |
|---|---|---|
| Histology[a] | MTU | 71 |
| | MTI | 52 |
| | MTT | 21 |
| | TD | 7 |
| | Unspecified NSGCT | 13 |
| Primary tumour site | Testis | 136 |
| | Retroperitoneal | 20 |
| | Mediastinal | 5 |
| | Unknown | 3 |
| Prognostic group (MRC) | Good | 57 |
| | Poor | 106 |
| | Not classifiable | 1 |
| Age | Median | 30 years |
| | Range | 14–83 years |
| Treatment centres | Large[b] | 90 |
| | Small | 74 |
| Primary chemotherapy[c] | PVB | 14 |
| | BEVIP | 4 |
| | POMB/ACE | 32 |
| | Cisplatin+etoposide+others | 114 |
| Number of courses | 2–3 | 7 |
| | 4 | 63 |
| | 5–6 | 52 |
| | >6 | 29 |
| | not known | 13 |
| Response to induction treatment | Complete response | 53 |
| | Incomplete response | 52 |
| | Partial response, marker negative | 48 |
| | Progressive disease | 11 |
| Total patients | | 164 |

[a]Pugh classification (see text). [b]>75 patients with metastatic non-seminoma in the original study (see text). [c]P: platinum, V: vinblastine, B: bleomycin, E: etoposide, I: ifosfamide; O: Oncovin, M: methotrexate, A: actinomycin D, C: cyclophosphamide.

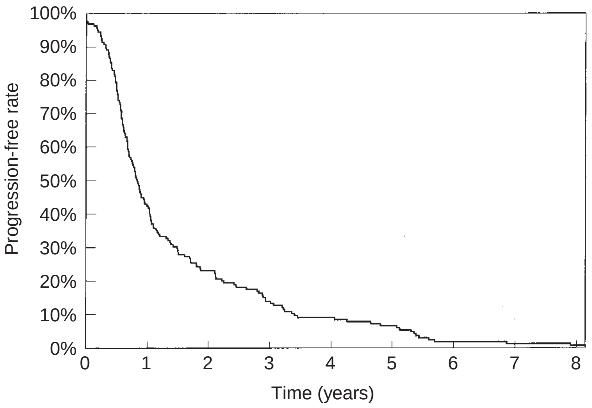

**Figure 1** Time from start of initial chemotherapy to relapse/progression

Few series have been published dealing with prognostic factors in patients with recurrent malignant germ cell tumours, and most are based on the experience of specialized institutions (Motzer et al, 1991; Horwich et al, 1993; Josefsen et al, 1993; Ledermann et al, 1994; Gerl et al, 1995). The aim of the present study was to establish prognostic factors based on a series of patients presenting to unselected oncological units. Such analyses may represent an appropriate background for the interpretation of the results of modern high-dose salvage chemotherapy and may also assist the clinician to identify future patients for risk-adapted salvage treatment.

### PATIENTS AND METHODS

In a previously published study prognostic factors were identified in 795 patients with advanced germ cell tumours (Mead et al, 1992). The present series represents a further analysis of those patients who progressed during or following cisplatin-based induction chemotherapy. This includes patients who never achieved a response and those with new disease activity after achieving a complete or partial response to primary chemotherapy. All patients had been treated with first-line platinum-based chemotherapy between 1982 and 1986.

As a rule patients with residual post-chemotherapy masses underwent surgery to remove them, and they received adjuvant cisplatin-based chemotherapy if residual germ cell malignancy was demonstrated in the post-chemotherapy resection specimen. Histological subtyping of the primary germ cell cancer was based on the Pugh Classification: MTU: malignant teratoma undifferentiated; MTI: malignant teratoma intermediate; MTT: malignant teratoma trophoblastic; TD: teratoma differentiated. In 13 patients with elevated serum AFP the malignant germ cell tumour could not be subtyped histologically.

For the present study all patients had data on baseline characteristics, and updated survival data. Seven centres provided further retrospective data on their patients and recorded details of the marker levels and sites of disease at progression. Based on the number of patients entered into the original series (Mead et al, 1992) ‘large oncological units’ were separated from ‘small’ ones. The former had contributed more than 75 patients with metastatic non-seminoma as opposed to < 75 for ‘small’ oncological units. This division was supported by patient entry by these centres into other Medical Research Council (MRC) trials. All patients in this study had received both induction and relapse chemotherapy at their local centre; patients referred to centres participating in this study for relapse treatment only were not included.

Complete response (CR) to treatment was defined as the clinical and radiological absence of all tumour manifestations (including normalization of serum alpha fetoprotein (AFP) – human chronic gonadotrophin (hCG), or the complete resection of residual mature teratoma or necrotic/fibrotic tumour tissue. Incomplete response (IR) comprised patients with persistently elevated markers (without serially rising values) or the histological demonstration of residual cancer in resection specimen. Patients with unresected residual tumour masses with normal tumour markers were included in the category Partial remission (PR) marker negative. Progression (PD) was defined as the development of new metastases and/or clearly rising serum tumour markers.

Factors predictive of survival from the date of progression were then identified. Potential prognostic factors included patient characteristics at initial diagnosis (site and extent of metastatic disease sites), response to initial chemotherapy, duration of relapse-free interval, and patient characteristics at relapse. An independent data set of 66 patients (provided by Dr A Gerl, Munich) was available on which to test the resulting prognostic models.

Survival times were measured from the date of diagnosis of progression to the date of death or date last seen. Survival curves were compared using the logrank test, and Cox’s proportional hazards regression model was used to identify independent

© 1999 Cancer Research Campaign

**Table 2** Univariate survival analysis from diagnosis of relapse

| Parameter | Number of patients | 2-Year survival rate | Log-rank | |
|---|---|---|---|---|
| | | | $\chi^{2b}$ | *P*-value[c] |
| Prognostic group at 1° diagnosis (MRC) | | | | |
| Good | 57 | 51% | 9.2 | 0.0024 |
| Poor | 106 | 32% | | |
| Site of primary tumour | | | | |
| Testis | 136 | 38% | 0.03 | 0.86 |
| Extragonadal | 28 | 41% | | |
| Age | | | | |
| <30 | 99 | 41% | 1.15 | 0.28 |
| 30–39 | 42 | 40% | | (t) |
| 40–49 | 13 | 31% | | |
| ≥50 | 10 | 20% | | |
| Response to induction treatment | | | | |
| Complete | 53 | 58% | 43.4 | ≤0.0001 |
| Incomplete | 52 | 27% | (3 d.f) | |
| PR marker negative | 48 | 37% | | |
| Progression | 11 | 0% | | |
| Time from initiation of primary therapy to progression (months) | | | | |
| <6 | 37 | 22% | 20.4 | <0.0001 |
| 6–12 | 57 | 28% | | (t) |
| 13–24 | 32 | 33% | | |
| 25–36 | 15 | 73% | | |
| >36 | 23 | 74% | | |
| hCG at relapse (IU $l^{-1}$)[a] | | | | |
| Normal | 57 | 39% | 1.40 | 0.24 |
| <100 | 23 | 48% | | (t) |
| 100–1000 | 19 | 32% | | |
| >1000 | 11 | 27% | | |
| AFP at relapse (kU $l^{-1}$)[a] | | | | |
| Normal | 55 | 45% | 3.65 | 0.056 |
| <100 | 33 | 37% | | (t) |
| 100–1000 | 13 | 23% | | |
| >1000 | 9 | 22% | | |
| Year of relapse treatment | | | | |
| Before 1986 | 94 | 29% | 7.15 | 0.008 |
| 1986–1991 | 70 | 51% | | |
| Treatment centre | | | | |
| 'Large' | 90 | 45% | 5.12 | 0.02 |
| 'Small' | 74 | 29% | | |
| Sites of relapse (1)* | | | | |
| Markers only | 16 | 38% | | |
| Abdominal nodes | 29 | 45% | | |
| Mediastinal/neck nodes (± abdo nodes) | 8 | 63% | 3.08 | 0.54 |
| Lung (± abdo/med/neck nodes) | 28 | 36% | (4 d.f) | |
| Other visceral mets (± other sites) | 29 | 28% | | |
| Sites of relapse (2)[a] | | | | |
| No lung or other visceral mets | 53 | 45% | 1.03 | 0.31 |
| Lung or other visceral mets present | 57 | 32% | | |

[a]Data available in a subgroup of 110 patients. [b]Chi square on 1 d.f. unless otherwise stated. [c](t) indicates chi square test for trend.

prognostic factors. A forward stepwise variable selection procedure was used.

### RESULTS

One hundred and sixty-four of the 795 patients (21%) relapsed. Of these, 116 (71%) have died and the median follow-up time of those still alive is 8.5 years (range 1–12.5 years). Patient characteristics at the time of initial diagnosis and details of primary chemotherapy are described in Table 1. The primary tumour site was identified in our study as the testis in 83% of the patients. Fifty-seven patients belonged at the start of induction chemotherapy to the good and 106 to the poor prognosis groups as defined by the previous MRC study (Mead et al, 1992). (One patient could not be classified.) The majority of patients received at least four courses of combination chemotherapy including both cisplatinum and etoposide. Twenty-one of the 164 patients had undergone adjuvant chemotherapy as histology of masses had demonstrated viable residual cancer.

Detailed data on characteristics at relapse were available for a subset of 110 patients, and these are included in Table 2. Relapse treatment was given between 1982 and 1991. In 103 patients a great variety of salvage regimens were employed, eighty-nine patients received cisplatin-based combinations, and no patient received high-dose chemotherapy. Ifosfamide-containing salvage

 *© 1999 Cancer Research Campaign*

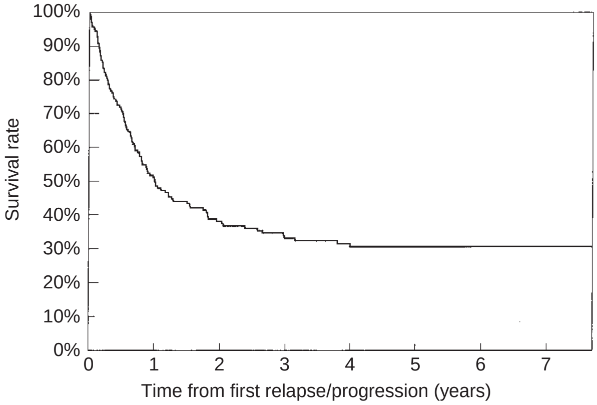

**Figure 2** Time from first relapse/progression to death

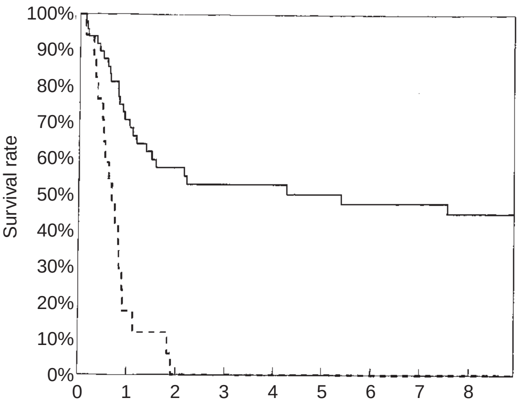

**Figure 4** Survival by prognostic group – test data set. (—) Good risk (≤ two risk factors) $n$ = 49; (- -) Poor risk (all three risk factors) $n$ = 17

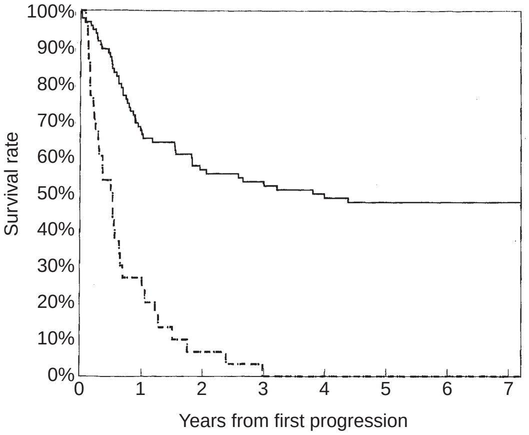

**Figure 3** Survival by prognostic group – developmental data set. (—) Good risk (≤ two risk factors) $n$ = 94; (- -) Poor risk (all three risk factors) $n$ = 30

chemotherapy was given to only 15 patients. No detailed information is available on the doses of the drugs and intervals between cycles. Thirty-two patients had surgery to remove residual masses in addition to chemotherapy. Seven patients did not receive salvage chemotherapy due to patient refusal (three patients), rapid death before start of chemotherapy (two patients), radiotherapy only (one patient) or surgery only (one patient). This latter patient had progressed with a 3-cm large retroperitoneal mass and raised AFP (48 kU $l^{-1}$) 15 months after discontinuation of primary chemotherapy. He was salvaged by surgery without further chemotherapy. The remaining six patients all died within 7 months of the diagnosis of their progression.

The role of surgery in addition to salvage chemotherapy was analysed specifically: of 31 patients in whom a residual mass was completely resected immediately before or after salvage chemotherapy, 16 are alive after 5 years. In 36 patients who after salvage chemotherapy had a complete radiological response, the 3-year survival rate was only 14%, whereas those with residual mass left unresected ($n$ = 32) had a 3-year survival rate of 31%.

The median time to progression from the start of initial induction chemotherapy (Figure 1) was 10 months (range 0–99 months) for all patients ($n$ = 164) and the subset with additional data on progression characteristics ($n$ = 110). Eleven patients progressed within 4 weeks from the date of the end of last chemotherapy cycle ('absolutely cisplatin refractory patients'). In 38 patients (23%) renewed tumour activity developed more than 2 years after the start of induction treatment. Nineteen of these 'late' relapses occurred among the 53 patients with a CR to induction treatment. Seven of the above 38 patients with progression > 2 years after induction chemotherapy occurred amongst 52 incompletely responding patients, and 12 patients with late progression were from 48 patients belonging to the PR marker negative category. Median survival time following progression was 1 year (Figure 2), and the 5-year survival rate for all patients following progression was 30% (95% confidence interval (CI) 23.4–37.6%). Ten progressing patients had increased serum markers more than 2 years after primary chemotherapy. With a minimum follow-up of 3 years, only three of these ten patients have died.

Table 2 gives the results of the univariate analyses. The most important factors indicating a poor prognosis were a short progression-free interval, less than complete response to induction chemotherapy (± surgery), poor prognostic group at diagnosis, as defined by MRC criteria (Mead et al, 1992), treatment for relapse commencing in the early 1980s and treatment given at a 'small' centre.

All the factors described in Table 2, together with age (as a continuous variable) and primary tumour site (testis vs extragonadal) were included in the multivariate analysis. Patients with missing data on any factor were excluded on a case-by-case basis. In addition, progression-free interval, AFP and hCG at the time of progression were considered using the categories defined in Table 2, and also using binary cut-points. The cut-points chosen separated complete responders from all other response categories, progression-free interval greater or less than 2 years, AFP at progression above or below 100 kU $l^{-1}$ and hCG at progression above or below 100 IU $l^{-1}$. A further binary variable was defined which identified patients with neither marker raised above these limits.

The multivariate analysis (Table 3) identified three factors of independent prognostic importance ($P$-value for inclusion < 0.1); progression-free interval, response to induction treatment and marker levels. The hazard ratios for patients with either IR or PR marker negative were very similar. The small group of six patients with PD had the poorest outcome. As the hazard ratios for the three above factors were dissimilar, a number of prognostic models were considered which applied different weightings to the

© 1999 Cancer Research Campaign 

**Table 3** Cox regression analysis: final model

| Variable | Regression coefficient (s.e.) | Hazard ratio |
|---|---|---|
| Time to progression | (reference category) | 1 |
| (≤ 2 vs > 2 years) | 0.80 (0.34) | 2.22 |
| High markers at relapse | (reference category) | 1 |
| AFP > 100 ku $l^{-1}$ and/or hCG > 100 IU $l^{-1}$) | 0.60 (0.25) | 1.82 |
| Response to induction treatment | | 1 |
| CR | (reference category) | |
| IR | 0.42 (0.31) | 1.52 |
| PR marker negative | 0.39 (0.31) | 1.49 |
| PD | 1.46 (0.48) | 4.28 |

**Table 4** Validation data summary: independent data set of 66 patients

| Characteristic | Group | Number of patients |
|---|---|---|
| Response to induction | CR to chemo ± surgery | 37 |
| treatment | Elevated markers/residual malignant tumour | 25 |
| | Residual mass unresected (markers normal) | 4 |
| Time to progression | ≤2 years | 53 |
| | >2 years | 13 |
| AFP at relapse (kμ $l^{-1}$) | Normal | 41 |
| | ≤ 100 | 7 |
| | >100 | 18 |
| hCG at relapse (IU $l^{-1}$) | Normal | 38 |
| | ≤ 100 | 11 |
| | >100 | 17 |
| Sites of relapse | | |
| Abdomen | No | 34 |
| | Yes | 32 |
| Lung | No | 43 |
| | Yes | 23 |
| Mediastinum | No | 61 |
| | Yes | 5 |
| Extrapulmonary | No | 42 |
| | Yes | 24 |
| Year of relapse treatment | 1980–1985 | 32 |
| | 1986–1990 | 21 |
| | 1990–1995 | 13 |
| Total patients | | 66 |

**Table 5** Efficacy of salvage treatment in (excluding high-dose regimens) in patients with malignant non-seminomatous germ cell tumours, progressing/relapsing after cisplatin-based induction chemotherapy

| 1st Author | Year | No. of patients | Chemotherapy schedule | CR rate | Long-term survival |
|---|---|---|---|---|---|
| Loehrer | 1986 | 48 | VIP[a,b] | 33% | NA[c] |
| Motzer | 1991 | 94 | Various[d] | 23% | 15% |
| Josefsen | 1993 | 55 | Various | NA | 23% |
| Horwich | 1993 | 105 | Various | NA | 35% |
| Ledermann | 1994 | 38 | Various[e] | 47% | 46% |
| Motzer | 1994 | 31 | Paclitaxel | 10% | NA |
| Gerl | 1995 | 67 | Various[f] | NA | 37% |
| McCaffrey | 1997 | 56 | VIP/VeIP | 36% | 40% |
| Loehrer | 1998 | 135 | VeIP | 50% | 46% |
| Fosså | Present series | 164 | Various | NA | 30% |

[a] V: VP-16, E: Etoposide, I: Ifosfamide, P: cis-platin, Ve: Vinblastine (Velbe[R]), B: Bleomycin. [b]After P Ve B. [c]Not available. [d]Mostly BEP after P Ve B. [e]Containing vincristine, MTX, actinomycin D. [f]Ifosfamide in 38 patients.

 *© 1999 Cancer Research Campaign*

various factors. As the overall survival for patients with and without information on markers at progression was very similar, patients without marker information were also included in the models where possible. The simplest model separated patients into two groups on the basis of the number of adverse risk factors they had. Given the three risk factors:

- progression-free interval < 2 years
- < CR to induction chemotherapy
- high markers at progression (AFP >100 kU $l^{-1}$ and/or hCG > 100 IU $l^{-1}$)

Patients with all three had a very poor prognosis; this group comprised 30 patients with a median survival time of 7 months and a 2-year survival rate of 7% (95% CI 0–15%). None of these patients survived beyond 3 years. Ninety-four patients (81 with data on all three factors and a further 13 without data on markers at relapse), that is those with at most two risk factors, formed a ‘good prognosis’ group with a 2-year survival rate of 56% (95% CI 46–66%), and a 5-year survival rate of 47% (37–56%). The survival curves for these two groups are shown in Figure 3. It was possible to subdivide the good prognosis group further, on the basis of progression-free interval – the 38 patients with a progression-free interval of more than 2 years had a 2-year survival rate of 74% (60–88%), while those with a shorter progression-free interval has a 2-year survival rate of 45% (32–58%).

### Model validation

The independent data set was used to test the prognostic model. The data included 66 patients with disease progression during or after platinum-based induction chemotherapy, none of whom received high-dose therapy on progression. Sixty patients received ‘conventional’ regimens for progressive germ cell malignancy (of whom 40 received cisplatin- and ifosfamide-based regimens with either vinblastine or etoposide), while six were treated with surgery and/or radiotherapy alone. Forty-three patients have died; of the 23 alive, median follow-up is 8 years (range 6 months to 14 years). The characteristics of these patients are described in Table 4, using the same criteria for response as defined for the principle data set collected by the MRC.

The two-group prognostic model was applied to this data, and the resulting survival curves are shown in Figure 4. The number of patients falling into the good and poor risk groups were 49 (74%) and 17 (26%) and the corresponding 2-year survival rates were 58% and 0% (43–72%), respectively. Five of the patients within the good risk group were categorized as ‘absolutely platinum refractory’, whereas this was the case for six patients within the poor prognosis group.

### DISCUSSION

### Progressive germ cell malignancy

Progression after IR/PR marker negative or relapse after CR was observed in 164 (21%) of the original 795 patients with malignant germ cell tumours treated with cisplatin-based induction chemotherapy. This percentage is comparable to other reports (Motzer et al, 1991; Horwich et al, 1993; Josefsen et al, 1993; Gerl et al, 1995). Although about 70% of the cases of progression occurred within the first year after the start of induction treatment, reactivation of the disease was observed in 23% patients after 2 years. This observation together with the possibility of cure in patients with ‘late’ progression indicate the necessity to continue regular follow-up in patients with metastatic germ cell tumours for at least 5 years and probably longer.

### Induction chemotherapy

Four 3-weekly cycles of BEP (bleomycin 90 mg $cycle^{-1}$, etoposide 500 mg $m^{-2}$ $cycle^{-1}$, cisplatin 100 mg $m^{-2}$ $cycle^{-1}$) are today considered to be the standard treatment with metastatic malignant germ cell tumour. The PVB (cisplatin, vinblastine, bleomycin) combination used during the early 1980s has been shown to be significantly inferior to the BEP combination both with regard to efficacy and toxicity (Williams et al, 1987), whereas ifosfamide-containing regimens have not proved to be superior as induction treatment (Nichols et al, 1995). Carboplatin-based induction chemotherapy is, however, less effective than BEP (Horwich et al, 1997). Failure of induction chemotherapy may be due to primary or secondary drug resistance or to insufficient dosing or incorrect drug selection. So far no study has addressed the role of induction chemotherapy as regards the outcome of salvage treatment (number of cycles, type, dose-intensity). In the present series data were not available to evaluate any inadequacy of the induction treatment in relapsing patients. In future studies concerning relapsing patients, information on type and intensity of the induction treatment should be provided, and analysed with regard to its prognostic significance.

### Salvage treatment

The present analysis concentrates on prognostic factors evaluable before salvage treatment. However, the importance of resection of residual masses in patients with recurrent germ cell malignancies is increasingly recognized (Cassidy et al, 1992). The present retrospective analysis supports this view, though the results have to be interpreted having selection bias in mind.

It was not possible to study the role of the different salvage chemotherapy regimens due to the considerable heterogeneity of the drugs and regimens used. Today ifosfamide- and vinblastine-containing combinations (Loehrer et al, 1998) would be those drugs most frequently selected in patients who have not received these agents during their induction treatment (or a methotrexate-containing regimen (Levi et al, 1990)). However, only 15 of our patients received ifosfamide. Recently, paclitaxel (Motzer et al, 1994) has been identified as an active drug for salvage chemotherapy of patients with relapsing germ cell tumours. The use of these drugs after a standard BEP regimen (bleomycin, etoposide, cisplatin) would, if at all, have increased the overall outcome of salvage treatment as compared to the present series, but with one or little impact on the presented prognostic pretreatment factors.

The overall long-term survival (and probably cure rate) for our progressing patients was 30% which is comparable to other reports (Table 5). On the other hand, McCaffrey et al’s (1997) survival curves visualize an almost 40% long-term overall survival rate in patients treated with ifosfamide-containing cisplatin-based salvage chemotherapy. A different composition of prognostic groups in large versus small institutions may be one reason for such differences. A further reason for varying results may be that contrary to McCaffrey et al’s report, we analysed our series according to the ‘intention to treat’ principle. This included five

*© 1999 Cancer Research Campaign*

patients who finally refused all treatment (three) or died before the start of salvage chemotherapy (two).

The use of high-dose chemotherapy with carboplatin, etoposide and ifosfamide together with haematopoietic stem cell support has been explored in single institution phase II studies (Broun et al, 1992; Siegert et al, 1994; Margolin et al, 1996). A multicentre experience has been analysed recently by Beyer et al (1996) Several of these studies have emphasized the role of patient selection and of prognostic factors. Beyer et al (1996) have determined independent poor prognosis parameters which made subgrouping of progressing patients possible with respectively 50%, 30% and 4% overall long-term survival after high-dose chemotherapy for treatment of first or subsequent relapse. These authors identified the following poor prognosis parameters: mediastinal non-seminomatous primary tumour, progressive disease before high-dose chemotherapy, disease refractory to conventional-dose cisplatin, hCG >1000 IU $l^{-1}$. Using conventional cisplatin-based salvage chemotherapy Gerl et al (1995), Josefsen et al (1993) and Motzer et al (1991) identified a complete response to induction treatment as an additional positive independent prognostic factor which is confirmed in the present study.

In contrast to the report by Nichols et al (1994) on relapsing patients (after CR) a long interval from induction therapy to first progression proved to be the most powerful prognostic parameter. This discrepancy is difficult to explain but may be related to slightly different selection of patients: our analysis include patients with primary IR/PR who progressed after > 2 years, whereas Nichols et al only discuss patients with initial CR. Baniel et al (1995) emphazised the role of surgery in patients with late relapses and suggested that chemotherapy was only limitedly effective in these patients, progressing after > 2 years. Our series does not allow an analysis concerning these aspects as the majority of evaluable patients underwent both surgery and chemotherapy. Our finding supported by data from Horwich et al (1993) although it should be noted that some of these patients were included in our data set also. Interestingly, raised markers in patients with a long progression-free interval were not an adverse feature, but this was the case when the progression-free survival was short.

The good prognosis group comprised 94 patients (76%) and had a 47% 5-year survival when treated with 'conventional' salvage chemotherapy. This survival rate is quite comparable with the results of high-dose chemotherapy followed by stem cell support which is increasingly being applied in progressing patients. Our observations thus strongly indicate the need for a randomized trial in these patients in order to prove any superiority of the high-dose chemotherapy approach. On the other hand, the survival in patients belonging to the poor prognostic group is dismal if 'conventional' salvage chemotherapy is applied. These patients should probably be offered more intensive treatment regimens than available in the 1980s, with the use of stem cell support if possible (Broun et al, 1992; Siegert et al, 1994; Beyer et al, 1996; Margolin et al, 1996).

The present observations are not directly comparable with the prognostic parameter analysis performed by Beyer et al (1996). Most of Beyer et al's patients had developed progressive disease twice when receiving high-dose chemotherapy. Beyer et al assessed survival from the time when high-dose treatment was given. The patients from the present series had progressed only once after start of induction chemotherapy, and their survival was calculated from the time of their first progression. In the present series all progressing patients were included in the analysis, even those who did not receive (planned) salvage chemotherapy, whereas all patients evaluated by Beyer et al had been treated with high-dose chemotherapy. Furthermore, as also pointed out by Beyer et al, 65 of their patients received high-dose chemotherapy while responding to conventional chemotherapy (even CR), whereas all our patients had progressed at commencement of salvage treatment. However, Beyer et al's results of salvage high-dose chemotherapy, although not directly comparable with the present series, represent a clear indication that high-dose chemotherapy may be effective in some patients who relapse despite conventional salvage treatment. This aspect will be further dealt with in a future case control study.

In conclusion, about 20% of the patients with metastatic non-seminoma are not cured by standard conventional induction chemotherapy. The overall 5-year survival rate for these patients is 30% after conventional cisplatin-based salvage chemotherapy used in a multicentre setting. The group consists of two prognostic subgroups: a good prognosis group (about 75%) with a 5-year survival rate of 47% and a poor prognosis group (about 25%) with no patient surviving after 3 years. The results from non-randomized new approaches of salvage therapy should be interpreted on the background of the above prognostic groups, keeping in mind that about one-third of the progressing patients may be cured by 'conventional' means.

### REFERENCES

Baniel J, Foster RS, Gonin R, Messemer JE, Donohue JP and Einhorn LH (1995) Late relapse of testicular cancer. *J Clin Oncol* **13**: 1170–1176

Beyer J, Kramar A, Mandanas R, Linkesch W, Greinix A, Droz JP, Pico JL, Diehl A, Bokemeyer C, Schmoll HJ, Nichols CR, Einhorn LH and Siegert W (1996) High-dose chemotherapy as salvage treatment in germ cell tumors: a multivariate analysis of prognostic variables [see comments]. *J Clin Oncol* **14**: 2638–2645

Broun ER, Nichols CR, Kneebone P, Williams SD, Loehrer PJ, Einhorn LH and Tricot GJ (1992) Long-term outcome of patients with relapsed and refractory germ cell tumors treated with high-dose chemotherapy and autologous bone marrow rescue. *Ann Intern Med* **117**: 124–128

Cassidy J, Lewis CR, Kaye SB and Kirk D (1992) The changing role of surgery in metastatic non-seminomatous germ cell tumour. *Br J Cancer* **65**: 127–129

Gerl A, Clemm C, Schmeller N, Hartenstein R, Lamerz R and Wilmanns W (1995) Prognosis after salvage treatment for unselected male patients with germ cell tumours. *Br J Cancer* **72**: 1026–1032

Horwich A, A'Hearn R, Gildersleve J and Dearnaley DP (1993) Prognostic factor analysis of conventional dose salvage therapy of patients with metastatic non-seminomatous germ cell cancer. *Proc Am Soc Clin Oncol* **12**: 232

Horwich A, Sleijfer DT, Fossa SD, Kaye SB, Oliver RT, Cullen MH, Mead GM, de Wit R, de Mulder PH, Dearnaley DP, Cook PA, Sylvester RJ and Stenning SP (1997) Randomized trial of bleomycin, etoposide, and cisplatin compared with bleomycin, etoposide, and carboplatin in good-prognosis metastatic nonseminomatous germ cell cancer: a Multiinstitutional Medical Research Council/European Organization for Research and Treatment of Cancer Trial. *J Clin Oncol* **15**: 1844–1852

Josefsen D, Ous S, Hoie J, Stenwig AE and Fossa SD (1993) Salvage treatment in male patients with germ cell tumours. *Br J Cancer* **67**: 568–572

Lederman GS and Garnick MB (1986) Possible benefit of doxorubicin treatment in patients with refractory germ cell cancer. *Cancer* **58**: 2393–2398

Ledermann JA, Holden L, Newlands ES, Begent RH, Rustin GJ, Bagshawe KD and Brampton M (1994) The long-term outcome of patients who relapse after chemotherapy for non-seminomatous germ cell tumours. *Br J Urol* **74**: 225–230

Levi JA, Thomson D, Harvey V, Gill G, Raghavan D, Tattersall M, Snyder R, Burns I, Sandeman T, Byrne M and Schwarz M (1990) Effective salvage chemotherapy with etoposide, dactinomycin and methotrexate in refractory germ cell cancer. *J Clin Oncol* **8**: 27–32

Loehrer PJ Sr, Einhorn LH and Williams SD (1986) VP-16 plus ifosfamide plus cisplatin as salvage therapy in refractory germ cell cancer. *J Clin Oncol* **4**: 528–536

 *© 1999 Cancer Research Campaign*

Margolin BK, Doroshow JH, Ahn C, Hamasaki V, Leong L, Morgan R, Raschko J, Shibata S, Somlo G and Tetef M (1996) Treatment of germ cell cancer with two cycles of high-dose ifosfamide, carboplatin, and etoposide with autologous stem-cell support [see comments]. *J Clin Oncol* **14**: 2631–2637

Loehrer PJ, Gonin R, Nichols CR, Weathers T and Einhorn LH (1998) Vinblastine plus ifosfamide plus cisplatin as initial salvage therapy in recurrent germ cell tumor. *J Clin Oncol* **16**: 2500–2504

McCaffrey JA, Mazumdar M, Bajorin DF, Bosl GJ, Vlamis V and Motzer RJ (1997) Ifosfamide-and cisplatin-containing chemotherapy as first-line salvage therapy in germ cell tumors: response and survival. *J Clin Oncol* **15**: 2559–2563

Mead GM, Stenning SP, Parkinson MC, Horwich A, Fossa SD, Wilkinson PM, Kaye SB, Newlands ES and Cook PA (1992) The Second Medical Research Council study of prognostic factors in nonseminomatous germ cell tumors. Medical Research Council Testicular Tumour Working Party [published erratum appears in *J Clin Oncol* 1992 **10**: 867]. *J Clin Oncol* **10**: 85–94

Motzer RJ, Geller NL, Tan CC, Herr H, Morse M, Fair W, Sheinfeld J, Sogani P, Russo P and Bosl GJ (1991) Salvage chemotherapy for patients with germ cell tumors. The Memorial Sloan-Kettering Cancer Center experience (1979–1989). *Cancer* **67**: 1305–1310

Motzer RJ, Bajorin DF, Schwartz LH, Hutter HS, Bosl GJ, Scher HI, Lyn P and Fischer P (1994) Phase II trial of paclitaxel shows antitumor activity in patients with previously treated germ cell tumors. *J Clin Oncol* **12**: 2277–2283

Nichols CR, Baniel J, Foster R, Donohue JP and Einhorn LH (1994) Late relapse of germ cell tumors. *Proc Am Soc Clin Oncol* **13**: 234

Nichols CR, Loehrer PJ, Einhorn LH, Propert K, Vogelzang NJ, Crawford ED, Sarosdy M and Trump D (1995) Phase III study of cisplatin, etoposide and bleomycin (PVP16B) or etoposide, ifosfamide and cisplatin (VIP) in advanced stage germ cell tumors; an intergroup trial. *Proc Am Soc Clin Oncol* **14**: 239

Siegert W, Beyer J, Strohscheer I, Baurmann H, Oettle H, Zingsem J, Zimmermann R, Bokemeyer C, Schmoll HJ and Huhn D (1994) High-dose treatment with carboplatin, etoposide, and ifosfamide followed by autologous stem-cell transplantation in relapsed or refractory germ cell cancer: a phase I/II study. The German Testicular Cancer Cooperative Study Group. *J Clin Oncol* **12**: 1223–1231

Williams SD, Birch R, Einhorn LH, Irwin L, Greco FA and Loehrer PJ (1987) Treatment of disseminated germ-cell tumors with cisplatin, bleomycin, and either vinblastine or etoposide. *N Engl J Med* **316**: 1435–1440

*© 1999 Cancer Research Campaign*
